# Supplementary material for: Comparative multiomics analysis of cell physiological state after culture in a basket bioreactor
Source: Sci Rep. 2022 Nov 23;12:20161. doi: 10.1038/s41598-022-24687-4 (PMC9686226; doi:10.1038/s41598-022-24687-4)
Supplement: Supplementary file 1 — Supplementary Information 1. [file 41598_2022_24687_MOESM1_ESM.zip › raw data/Transcriptomic raw data/4.Quant/2.Correlation/PCA3D.pdf]

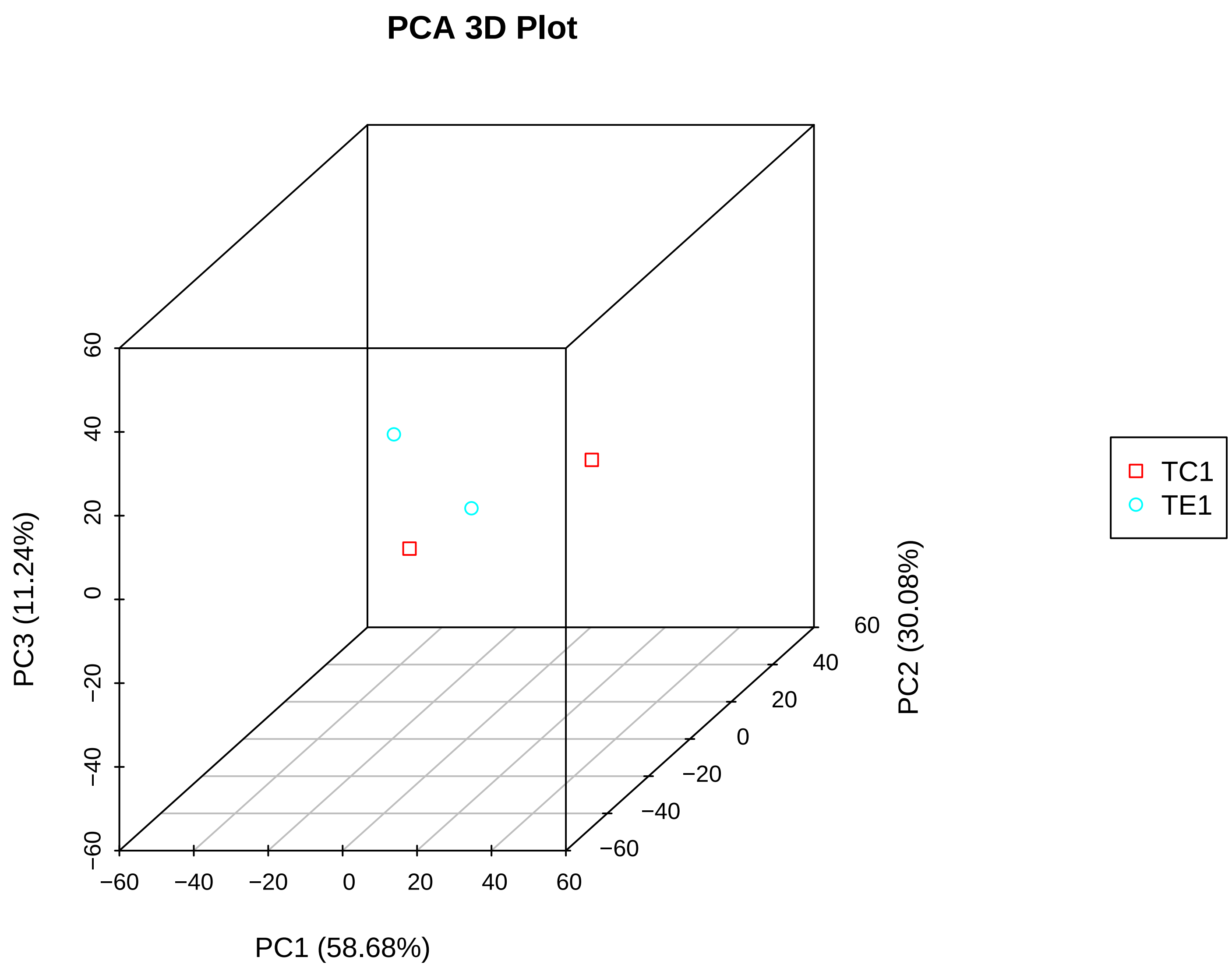

PCA 3D Plot
PC1 (58.68%)
PC2 (30.08%)
PC3 (11.24%)
−60
−40
−20
0
20
40
60
TC1
TE1
